# Supplementary material for: A Global Screen for Assembly State Changes of the Mitotic Proteome by SEC-SWATH-MS
Source: Cell Syst. 2020 Feb 26;10(2):133–155.e6. doi: 10.1016/j.cels.2020.01.001 (PMC7042714; doi:10.1016/j.cels.2020.01.001)

Q8TD19 | NEK9_HUMAN | NEK9 KIAA1995 NEK8 NERCC
Monomer MW [kDa]: 107.168 Monomer expected elution fraction: 40

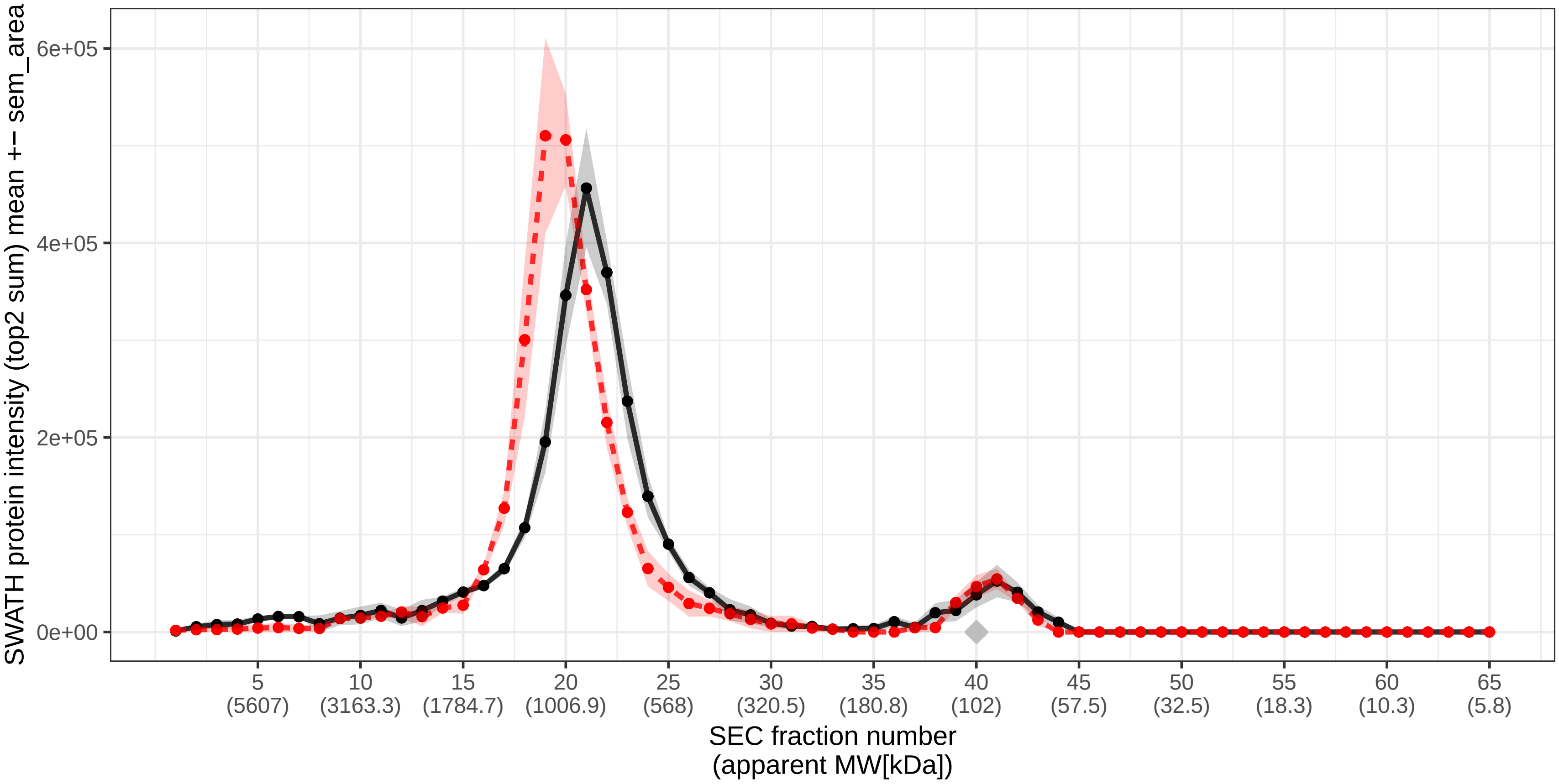

Supplement: Data S1. SEC-SWATH-MS Protein Chromatograms, Related to Figure 1 [file mmc6.zip › SECchrom_Q8TD19_NEK9_HUMAN_NEK9_KIAA1995_NEK8_NERCC.pdf]
